# Genome-wide association analysis of total cholesterol and high-density lipoprotein cholesterol levels using the Framingham Heart Study data

**DOI:** 10.1186/1471-2350-11-55

**Published:** 2010-04-06

**Authors:** Li Ma, Jing Yang, H Birali Runesha, Toshiko Tanaka, Luigi Ferrucci, Stefania Bandinelli, Yang Da

**Affiliations:** 1 Department of Animal Science, University of Minnesota, USA; 2 Supercomputer Institute, University of Minnesota, USA; 3 Medstar Health Research Institute, Baltimore MD, USA; 4 Longitudinal Study Section, National Institute on Aging, Baltimore MD, USA; 5 Geriatric Unit, Azienda Sanitaria Firenze (ASF), Florence, Italy

## Abstract

**Background:**

Cholesterol concentrations in blood are related to cardiovascular diseases. Recent genome-wide association studies (GWAS) of cholesterol levels identified a number of single-locus effects on total cholesterol (TC) and high-density lipoprotein cholesterol (HDL-C) levels. Here, we report single-locus and epistasis SNP effects on TC and HDL-C using the Framingham Heart Study (FHS) data.

**Results:**

Single-locus effects and pairwise epistasis effects of 432,096 SNP markers were tested for their significance on log-transformed TC and HDL-C levels. Twenty nine additive SNP effects reached single-locus genome-wide significance (p < 7.2 × 10^-8^) and no dominance effect reached genome-wide significance. Two new gene regions were detected, the *RAB3GAP1-R3HDM1-LCT-MCM6 *region of chr02 for TC identified by six new SNPs, and the *OSBPL8-ZDHHC17 *region (chr12) for HDL-C identified by one new SNP. The remaining 22 single-locus SNP effects confirmed previously reported genes or gene regions. For TC, three SNPs identified two gene regions that were tightly linked with previously reported genes associated with TC, including rs599839 that was 10 bases downstream *PSRC1 *and 3.498 kb downstream *CELSR2*, rs4970834 in *CELSR2*, and rs4245791 in *ABCG8 *that slightly overlapped with *ABCG5*. For HDL-C, *LPL *was confirmed by 12 SNPs 8-45 kb downstream, *CETP *by two SNPs 0.5-11 kb upstream, and the *LIPG-ACAA2 *region by five SNPs inside this region. Two epistasis effects on TC and thirteen epistasis effects on HDL-C reached the significance of "suggestive linkage". The most significant epistasis effect (p = 5.72 × 10^-13^) was close to reaching "significant linkage" and was a dominance × dominance effect of HDL-C between *LMBRD1 *(chr06) and the *LRIG3 *region (chr12), and this pair of gene regions had six other D × D effects with "suggestive linkage".

**Conclusions:**

Genome-wide association analysis of the FHS data detected two new gene regions with genome-wide significance, detected epistatic SNP effects on TC and HDL-C with the significance of suggestive linkage in seven pairs of gene regions, and confirmed some previously reported gene regions associated with TC and HDL-C.

## Background

Total cholesterol (TC) is related to coronary diseases and high-density lipoprotein (HDL-C) cholesterol is anti-atherogenic. Genome-wide association studies (GWAS) and human genetic studies have identified a number of genes and gene regions affecting cholesterol phenotypes including TC and HDL-C [1-11]. A meta-analysis of HDL-C levels that include the FHS data has previously been published [2]. An early report on FHS [12] analyzed TC and HDL-C but used 100 k SNPs and a sample size that was much smaller than the current FHS sample size. Epistasis analysis of TC and HDL-C was unavailable. Here, we apply a quantitative genetics approach to detect additive or dominance single-locus effects and epistasis effects on log-transformed TC and HDL-C using 432,096 SNP markers and over 6000 individuals in FHS. The epistasis effects we tested included additive × additive (A × A), additive × dominance (A × D) or dominance × additive (D × A), and dominance × dominance (D × D) effects, with genetic interpretations of allele × allele, allele × genotype or genotype × allele, and genotype × genotype interactions. The single-locus analysis was intended to detect new targets or confirm existing targets using a method of analysis different from those used in previous reports based on an extended Kempthorne model that allows Hardy-Weinberger disequilibrium and linkage disequilibrium [13] for GWAS analysis of the FHS data while the epistasis analysis of TC and HDL-C was the first such attempt using the FHS data and the 500 k SNP panel.

## Results

The single-locus tests detected nine SNPs with additive (or allelic) effects on TC and twenty SNPs with additive effects on HDL-C that reached genome-wide significance (Tables 1-2). No dominance effect reached genome-wide significance. Among the twenty nine SNP effects, twenty were new effects that were not reported in previous studies and nine were previously reported to be associated with various cholesterol phenotypes [1-12]. Seven SNPs identified two new gene regions while the remaining twenty two SNPs confirmed previously reported gene regions. Two epistasis effects on TC and thirteen epistasis effects on HDL-C representing seven pairs of gene regions reached the significance of "suggestive linkage".

**Table 1:** Single-locus SNP effects for TC with genome control (GC) adjusted P < 7.2 × 10^-8^.

						Effect Type & P value		
							
SNP	Chr	Position	Gene Region	Reported SNP effect	MAF	Genotype	Additive	Effect Size
rs4970834	1	109814880	*CELSR2*^a ^(intron 28)	Non-HDL-C [5-7]	0.18	1.10E-08	1.75E-09	0.146 ± 0.023
				TC [8]				
rs599839	1	109822166	10 bases downstream	LDL-C [6,7,9,10]	0.22	8.72E-14	2.46E-14	0.174 ± 0.021
			*PSRC1*^a^	Non-HDL-C [5]				
rs4245791	2	44074431	*ABCG8 *(intron 3)		0.32	1.82E-07	3.33E-08	-0.127 ± 0.021
rs6730157	2	135907088	*RAB3GAP1 *(intron 17)	LDL-C: P = 0.018 [2]^b^	0.45	8.51E-08	2.16E-08	0.113 ± 0.019
rs12465802	2	136381348	*R3HDM1 *(intron 7)	LDL-C: P = 0.022 [2]^b^	0.44	2.63E-08	7.98E-09	0.117 ± 0.019
rs4954280	2	136420690	*R3HDM1 *(intron18)	LDL-C: P = 0.007 [2]^b^	0.33	1.49E-07	5.87E-08	0.114 ± 0.02
rs2322660	2	136557319	*LCT *(intron 12)	LDL-C: P = 0.055 [2]^b^	0.35	2.42E-08	7.08E-09	-0.120 ± 0.019
				TC: P = 0.003-0.005 [17]				
				LDL-C: P = 0.002-0.0005 [17]				
rs309180	2	136614255	*MCM6 *(intron 11)	LDL-C: P = 0.057 [2]^b^	0.36	2.43E-08	8.39E-09	-0.119 ± 0.019
rs632632	2	136638216	4.2 kb upstream *MCM6*	LDL-C: P = 0.216 [2]^b^	0.36	2.50E-08	1.03E-08	-0.118 ± 0.019

^a ^This gene was reported to be associated with TC [1].

^b ^Available at http://www.sph.umich.edu/csg/abecasis/public/lipids2008/

**Table 2:** Single-locus SNP effects for HDL-C with genome control (GC) adjusted P < 7.2 × 10^-8^.

						**Effect Type & P value**		
							
**SNP**	**Chr**	**Position**	**Gene Region**	**Reported SNP effect**	**MAF**	**Genotype**	**Additive**	**Effect Size**
rs17482753	8	19832646	8 kb downstream *LPL*^a^	Triglyceride [7]	0.10	1.27E-08	3.50E-09	-0.191 ± 0.031
rs10503669	8	19847690	23 kb downstream *LPL*^a^	HDL-C [4,11]	0.09	3.75E-08	1.14E-08	-0.189 ± 0.031
rs17410962	8	19848080	23 kb downstream *LPL*^a^		0.12	2.75E-08	4.27E-09	0.173 ± 0.028
rs17489268	8	19852045	27 kb downstream *LPL*^a^		0.27	2.28E-10	5.97E-11	0.141 ± 0.02
rs17411031	8	19852310	28 kb downstream *LPL*^a^	HDL-C [7]	0.27	3.06E-10	7.21E-11	-0.14 ± 0.02
rs17489282	8	19852518	28 kb downstream *LPL*^a^		0.25	2.33E-09	6.54E-10	0.14 ± 0.021
rs4922117	8	19852586	28 kb downstream *LPL*^a^		0.25	2.28E-09	7.73E-10	-0.124 ± 0.019
rs17411126	8	19855272	31 kb downstream *LPL*^a^		0.27	6.14E-10	1.23E-10	0.138 ± 0.02
rs765547	8	19866274	42 kb downstream *LPL*^a^		0.27	1.60E-10	3.07E-11	-0.142 ± 0.02
rs11986942	8	19867445	43 kb downstream *LPL*^a^		0.33	2.22E-07	5.53E-08	0.112 ± 0.02
rs1837842	8	19868290	44 kb downstream *LPL*^a^		0.27	2.14E-10	4.09E-11	0.142 ± 0.02
rs1919484	8	19869676	45 kb downstream *LPL*^a^		0.27	4.12E-10	7.21E-11	-0.143 ± 0.021
rs17259942	12	77072077	*OSBPL8*-*ZDHHC17*		0.12	8.61E-08	1.81E-08	0.168 ± 0.028
rs9989419	16	56985139	*HERPUD1*-*CETP*^a^	HDL-C [4,7]	0.40	4.57E-13	5.96E-14	-0.147 ± 0.019
rs1800775	16	56995236	0.5 kb upstream *CETP*^a^	HDL-C [3,12]	0.45	1.54E-29	1.64E-30	0.242 ± 0.02
rs7240405	18	47159090	*LIPG*^a^-*ACAA2*^b^		0.16	8.01E-08	1.58E-08	-0.145 ± 0.024
rs4939883	18	47167214	*LIPG*^a^-*ACAA2*^b^	HDL-C [1,2]	0.17	1.07E-07	1.85E-08	0.142 ± 0.024
rs1943981	18	47169815	*LIPG*^a^-*ACAA2*^b^		0.17	1.49E-07	2.50E-08	0.142 ± 0.024
rs2156552	18	47181668	*LIPG*^a^-*ACAA2*^b^	HDL-C [3,4]	0.16	5.87E-08	1.14E-08	-0.146 ± 0.024
rs6507945	18	47243912	*LIPG*^a^-*ACAA2*^b^		0.43	4.34E-08	6.91E-09	0.113 ± 0.018

^a ^This gene was reported in [1-4].

^b ^This gene was reported in [3].

### Single-locus effects

**Figure 1:**
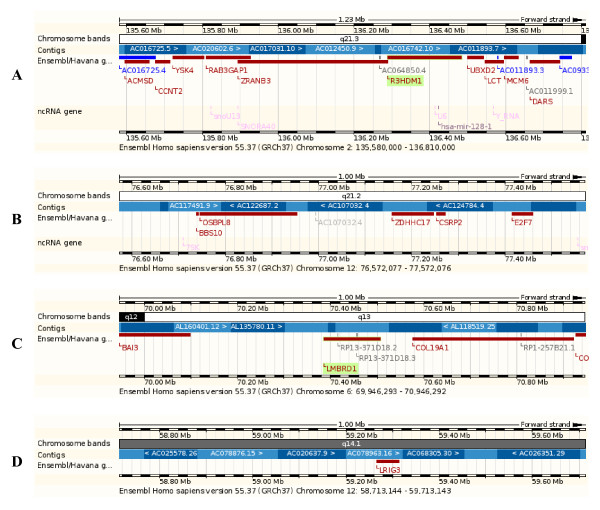
**Gene regions associated with total cholesterol (TC)**. **A**) A 1.23 Mb region containing *RAB3GAP1-R3HDM1-LCT-MCM6 *with multiple SNP effects on TC. **B**) One Mb region containing *OSBPL8-ZDHHC17 *associated with HDL-C. **C**) One Mb region containing *LMBRD1 *that had multiple SNPs interacting with an SNP near *LRIG3 *for TC. **D**) One Mb region containing *LRIG3 *which was near an SNP interacting with multiple SNPs in *LMBRD1 *for TC.

For TC, nine SNPs with additive (or allelic) effects reached genome-wide significance with p < 7.2 × 10^-8 ^(Table 1). Six SNPs inside or near four genes identified a new chr02 region containing *RAB3GAP1*, *R3HDM1*, *LCT *and *MCM6 *to be associated with TC (Figure 1A). Of the six SNPs in the *RAB3GAP1-R3HDM1-LCT-MCM6 *region, five SNPs were inside genes and one SNP was 4.2 kb upstream *MCM6*. The most significant SNP in this region was rs2322660 in intron 12 of *LCT *(Table 1). The *RAB3GAP1-R3HDM1-LCT-MCM6 *region contained two other genes (*ZRANB3 *and *UBXD2*) that did not have significant SNPs. Eleven other SNPs spanning a 1.23 Mb region (Figure 1A) that includes *RAB3GAP1-R3HDM1-LCT-MCM6 *had p-values between 1.27 × 10^-5 ^and 7.13 × 10^-7^, including one SNP upstream *ACMSD*, one SNP in *ACMSD*, two SNPs in *YSK4*, one SNP in *R3HDM1*, one SNP in *UBXD2 *(also named *UBXN4 *according to NCBI [14]), two SNPs in *LCT*, and three SNPs downstream *DARS *(data not shown). These less significant results in the same neighborhood should add to the significance of the *RAB3GAP1-R3HDM1-LCT-MCM6 *region to TC. Three SNPs identified two genes that were tightly linked with previously reported genes associated with TC [1]. These three SNPs were rs599839 that was 10 bases downstream *PSRC1 *(chr01) and 3.498 kb downstream *CELSR2*, rs4970834 in intron 28 of *CELSR2*, and rs4245791 in intron 3 of *ABCG8 *(chr02) that slightly overlapped with *ABCG5*, where *CELSR2 *and *ABCG5 *regions were reported to be associated with TC in a recent GWAS report [1]. *PSRC1 *and *ABCG8 *also were reported to affect low-density lipoprotein cholesterol (LDL-C) [2-4,6,7,9,10]. The SNP (rs599839) that was 10 bases downstream *PSRC1 *had the most significant single-locus effect on TC (p = 3.7 × 10^-16^), while the SNP inside *CELSR2 *(rs4970834) had the second most significant single-locus effect on TC (p = 1.29 × 10^-10^).

For HDL-C, twenty SNPs with additive effects reached genome-wide significance (Table 2). SNP rs17259942 identified a new gene region associated with HDL-C, the *OSBPL8-ZDHHC17 *region (q21.2, Figure 1B), with rs17259942 being 117 kb downstream *OSBPL8 *and 85 kb upstream *ZDHHC17 *[15]. According to NCBI [14], the *OSBPL8-ZDHHC17 *region contained three pseudo-genes (*RPL7AP59*, *RPL21P98 *and *RPL7P43*) and rs17259942 was 18 kb downstream *RPL21P98 *and 43 kb upstream *RPL7P43*. The other nineteen SNPs confirmed previously reported gene regions, including twelve SNPs 8-45 kb downstream *LPL*, two SNPs 0.5-11 kb upstream *CETP*, and five SNPs in the *LIPG-ACAA2 *region (39.812 kb downstream *LIG *and 65.963 kb upstream *ACAA2*) [1-4,7,11,12]. *LPL*, *CETP *and *LIPG *were reported to be associated with HDL-C in four recent GWAS reports [1-4] while *ACAA2 *was reported in [3]. The SNP nearest to *CETP *(rs1800775) was the most significant effect (p = 8.61 × 10^-34^) in this study.

**Figure 2:**
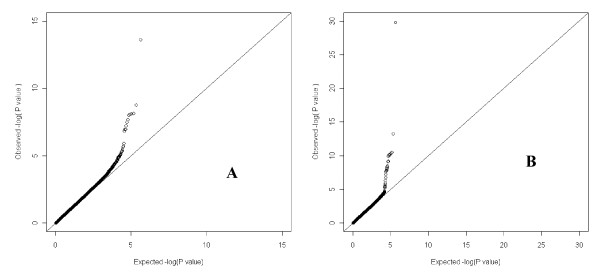
**QQ plots for single-SNP whole genome association tests of total cholesterol (TC) and high-density lipoprotein cholesterol (HDL-C)**. **A**) TC. **B**) HDL-C.

QQ plot for single SNP tests on TC and HDL-C showed that p-values of significant results all deviated from the expected p-values under the null hypothesis (Figure 2).

### Epistasis effects

Two epistasis effects on TC and thirteen epistasis effects on HDL-C reached the significance of suggestive linkage defined in [16] (Table 3). The two epistasis effects on TC involved two different pairs of gene regions while the thirteen epistasis effects on HDL-C involved five different pairs of gene regions, so that the fifteen epistasis effects identified seven pairs of gene regions. Eight SNPs in introns 1, 5, 7, 9, and 14 of *LMBRD1 *(chr06) (Figure 1C) interacted with a chr12 SNP about 53 kb from *LRIG3 *(q14.1, Figure 1D) and all these eight pairs had D × D effects on HDL-C. One of the eight epistasis effects involving intron 14 of *LMBRD1 *was the most significant epistasis effect that was close to reaching "significant linkage" defined in [16] or genome-wide significance with 5% Bonferroni corrected type-I error.

**Table 3:** Epistasis effects for TC and HDL-C with the significance of suggestive linkage.

										P value			
												
SNP1	Chr1	Pos1	Gene1	MAF1	SNP2	Chr2	Pos2	Gene2	MAF2	Genotype	Epistasis		Effect
**TC**													

rs4437278	4	12488199	*U6*^a ^(174 kb)	0.15	rs705169	10	125285443	*GRP26*(140 kb)	0.49	4.49E-10	AA	5.93E-12	0.249 ± 0.036
rs4738150	8	72607907	*U8*^a ^(120 kb)	0.40	rs16918936	9	33009027	*APTX*, *LOC646808*	0.04	4.68E-13	AD	1.29E-12	-1.348 ± 0.188

**HDL-C**													

rs10476539	5	91991628	*AC026781.5*^a ^(62 kb)	0.18	rs2392885	8	129003117	*PVT1*^a^	0.28	2.63E-10	AA	2.99E-12	-0.269 ± 0.037
rs4706271	6	70390132	*LMBRD1*(intron14)	0.41	rs6581219	12	59213144	*LRIG3*(53 kb)	0.42	7.67E-11	DD	5.72E-13	-0.412 ± 0.056
rs7741758	6	70412380	*LMBRD1*(intron9)	0.41	rs6581219	12	59213144	*LRIG3*(53 kb)	0.42	5.73E-10	DD	4.60E-12	-0.388 ± 0.054
rs9346333	6	70426479	*LMBRD1*(intron8)	0.41	rs6581219	12	59213144	*LRIG3*(53 kb)	0.42	1.39E-10	DD	1.16E-12	-0.398 ± 0.054
rs9351772	6	70428200	*LMBRD1*(intron8)	0.41	rs6581219	12	59213144	*LRIG3*(53 kb)	0.42	5.38E-10	DD	4.25E-12	-0.390 ± 0.055
rs7762400	6	70445634	*LMBRD1*(intron7)	0.41	rs6581219	12	59213144	*LRIG3*(53 kb)	0.42	4.41E-10	DD	3.52E-12	-0.388 ± 0.054
rs9294851	6	70457629	*LMBRD1*(intron5)	0.41	rs6581219	12	59213144	*LRIG3*(53 kb)	0.42	2.83E-10	DD	2.22E-12	-0.392 ± 0.054
rs9354890	6	70504296	*LMBRD1*(intron1)	0.41	rs6581219	12	59213144	*LRIG3*(53 kb)	0.42	5.54E-10	DD	4.18E-12	-0.388 ± 0.054
rs9364063	6	70514750	*LMBRD1*(8 kb)	0.41	rs6581219	12	59213144	*LRIG3*(53 kb)	0.42	4.65E-10	DD	4.46E-12	-0.386 ± 0.054
rs2787520	6	106821428	*ATG5*(48 kb)	0.35	rs7236739	18	20800715	*CABLES1*(intron4)	0.31	2.98E-10	AA	2.48E-12	-0.211 ± 0.029
rs2842169	10	128330713	*AL583860.7*^a ^(85 kb)	0.10	rs4756344	11	36765284	*C11orf74*(84 kb)	0.26	3.56E-10	AA	3.73E-12	0.348 ± 0.049
rs17623128	16	77630108	*AC092724.2*^a ^(114 kb)	0.33	rs6506699	18	9775566	*RAB31*	0.49	4.65E-10	DD	2.78E-12	-0.449 ± 0.062
rs12596869	16	77630380	*AC092724.2*^a ^(114 kb)	0.33	rs6506699	18	9775566	*RAB31*	0.49	3.81E-10	DD	2.30E-12	-0.451 ± 0.062

^a ^This was an RNA gene.

Among the seven different pairs of gene regions with epistasis effects, four pairs had A × A effects, one pair had A × D effect, and two pairs had D × D effects (Table 3). For the A × A effect on TC involving chr04 and chr10, the A-T gamete had the highest TC value while the A-G gamete had the lowest TC value (Table 4). This showed that the G and T alleles of rs705169 on chr10 had significantly different effects when combined with the A allele of rs4437278 on chr04, noting that rs705169 did not have significant single-locus effect. The same phenomenon was also observed for the other three A × A effects in Table 4. For the A × D effect, the A-GG allele-genotype combination had the highest TC value while the G-GG allele-genotype combination had the lowest TC value. The two D × D effects were on HDL-C. For the D × D effect of rs4706271 × rs6581219 representing the eight pairs of D × D effects involving the same gene regions, GT-GG had the highest HDL-C value while GG-GG had the lowest HDL-C value. For the remaining D × D effect of rs12596869 × rs6506699 representing the two D × D effects of the same gene regions, CC-AG had the highest HDL-C value while CC-AA had the lowest HDL-C value (Table 4).

**Table 4:** Frequency and effect of gamete, allele-genotype or genotype-genotype combination in each epistasis effect with statistical significance of suggestive linkage.

Trait	SNP1	Chr1	SNP2	Chr2										
					Gamete	A-T	G-G	G-T	A-G					
	rs4437278	4	rs705169	10	Frequency	0.0785	0.413	0.434	0.0742					
TC					Effect	0.103	0.0195	-0.0186	-0.108					
					Allele-genotype	A-GG	G-GT	A-TT	G-TT	A-GT	G-GG			
	rs4738150	8	rs16918936	9	Frequency	0.0015	0.0284	0.557	0.374	0.0387	0.001			
					Effect	0.961	0.0917	0.00219	-0.00313	-0.0636	-1.42			

					Gamete	A-C	G-T	G-C	A-T					
	rs10476539	5	rs2392885	8	Frequency	0.0538	0.593	0.226	0.127					
					Effect	0.15	0.0137	-0.0359	-0.0635					
					Genotype-genotype	GT-GG	GG-AG	GT-AA	TT-AG	TT-AA	TT-GG	GT-AG	GG-AA	GG-GG
	rs4706271	6	rs6581219	12	Frequency	0.0817	0.0887	0.161	0.169	0.112	0.0619	0.24	0.055	0.0298
					Effect	0.137	0.127	0.0694	0.066	-0.0495	-0.0908	-0.0932	-0.103	-0.189
					Gamete	G-G	T-A	G-A	T-G					
HDL-C	rs2787520	6	rs7236739	18	Frequency	0.106	0.448	0.241	0.206					
					Effect	0.0946	0.0224	-0.0416	-0.0488					
					Gamete	C-A	T-G	T-A	C-G					
	rs2842169	10	rs4756344	11	Frequency	0.0751	0.237	0.661	0.0272					
					Effect	0.0801	0.0253	-0.00908	-0.221					
					Genotype	CC-AG	CT-AA	CT-GG	TT-AG	TT-GG	TT-AA	CT-AG	CC-GG	CC-AA
	rs12596869	16	rs6506699	18	Frequency	0.0501	0.103	0.115	0.208	0.131	0.115	0.215	0.0344	0.0279
					Effect	0.223	0.109	0.0979	0.0535	-0.0433	-0.0494	-0.103	-0.167	-0.197

## Discussion

The single-locus results in this study had strong confirmations with existing studies. For TC, we confirmed *CELSR2 *and *ABCG5 *reported in [1,8]. These confirmed TC results should be considered as strong confirmation because our study had no overlapping samples with studies of [1,8]. We detected seven effects on TC in the *RAB3GAP1-R3HDM1-LCT-MCM6 *region with the SNP in *LCT *being the most significant. Six of these seven effects had p-values for LDL-C in the range of 0.007-0.056 from a meta-analysis ([2], Table 1). This could be an indication about the significance on TC from a meta-analysis because LDL-C is calculated from TC [17]. A study in FINRISK cohorts with 14,140 individuals reported that *LCT *was associated with both TC and LDL-C with p-values in the range of 0.0005-0.005 [18]. *In Silico *replication using 1231 Italian subjects from the InCHIANTI cohort [19] generally lacked confirmation for the TC results in Table 1. The first three markers had p-values in the range of 0.005-0.07 while the other effects had p-values greater than 0.14 from the InCHIANTI cohort. The biological function of *LCT *for digesting lactose could be a reason for agreements and disagreements in replicating *LCT *effects on cholesterol. *LCT *affects lactose digestion and long-term consumption of lactose in rats was found to affect aortic cholesterol levels [20]. Therefore, dietary lactose levels that have not been considered by human GWAS could have affected the *LCT *results of different studies. *MCM6 *contains two of the regulatory regions for *LCT *[21] so that the significant effects in or near *MCM6 *(Table 1) could be due to *MCM6*'s regulatory role to *LCT*. HDL-C had twenty significant SNP effects, but only one SNP identified a new gene region (*OSBPL8-ZDHHC17*) while all the other SNPs confirmed previously reported gene regions, although only seven of the twenty significant SNPs for HDL-C were reported previously (Table 2). *OSBPL8 *encodes a group of intracellular lipid receptors and suppresses *ABCA1 *[22], and *ABCA1 *was found to affect HDL-C level [23,24]. For HDL-C, the InCHIANTI cohort did not confirm the effects in the *LIPG-ACAA *region (p > 0.55) but confirmed the other effects. In light of different samples and different methods of data analysis between our study and those in previous reports, the confirmations of gene results we observed for TC and HDL-C should be considered strong confirmations. This study used log-transformed TC and HDL-C values while recent GWAS on TC [1] and HDL-C [1-4] used the original observations of TC and HDL-C that deviated from normal distribution. However, single-locus effects from our study and previous studies [1-4] had remarkable mutual confirmation, indicating that single-locus analysis was somewhat robust to data distribution and possibly to methods of analysis.

Epistasis effects on TC and HDL-C were not reported in other GWAS so that a comparison between our epistasis results and those from others was unavailable. We detected eight SNP pairs indicating the interaction between gene *LMBRD1 *and gene *LRIG3 *with the significance of suggestive linkage. Both *LMBRD1 *and *LRIG3 *encode membrane proteins. *LMBRD1 *gene is involved in the transportation and metabolism of vitamin B12 which is important for metabolism of branched chain amino acids and odd chain fatty acids [25]. Replication using the InCHIANTI cohort did not confirm the epistasis results (p > 0.15).

The statistical power of epistasis testing is less than that for testing a single-locus effect, particularly for epistasis effects involving dominance such as A × D and D × D effects, with D × D effect being the most difficult to detect. The reason for this difficulty was due to the fact that higher-order effects explain less phenotypic variation even if the effect sizes were the same as lower-order effects [13]. The reduced power for epistasis testing could have contributed to the fact that the epistasis effects we detected only reached 'suggestive linkage' although the sample size was over 6000. The data analysis of this study showed that pairwise analysis was sensitive to outliers. This was due to the fact that artificially significant epistasis effects could occur when rare combinations of loci had extreme genotypic values by chance. This may happen when outliers exist due to the large number of pairwise effects arising from the large number of pairwise combinations. For example, over 466 billion pairwise effects (93,353,260,560 pairs × 5 effects per pair = 466,766,302,800 pairwise effects) were tested per trait in this study. A small fraction of random association between rare frequencies and outliers in opposite directions among a large number of pairs could yield a long list of artificially significant epistasis results. Therefore, dealing with outliers such as removing outliers and using data transformation is important in pairwise analysis. Pairwise analysis is computationally intensive but timely analysis is possible using parallel computing. Using 784 processor cores on the SGI Altix XE 1300 Linux cluster system with 2.66 GHz Intel Clovertown processor at the Minnesota Supercomputer Institute, the completion of pairwise epistasis analysis required about 15 hours per trait.

## Conclusions

Genome-wide association analysis of the FHS data detected new single-locus and epistasis effects on TC and HDL-C and confirmed some previously reported effects. Additive effects were the primary single-locus effects of TC and HDL-C while epistasis effects involved allele × allele, allele × genotype (or genotype × allele), and genotype × genotype interactions.

## Methods

### Phenotype and SNP data

The FHS GWAS data (version 2) had 6575 individuals with SNP genotypes of the 500 k SNP panel from dbGAP. Of the 6575 individuals, 6431 had observations on TC and 6078 individuals had observations on HDL-C. A total of 496,858 SNP markers had known chromosome locations and 432,096 of these SNP markers with minor allele frequencies greater than or equal to 0.01 were analyzed.

### Statistical Analysis

**Figure 3:**
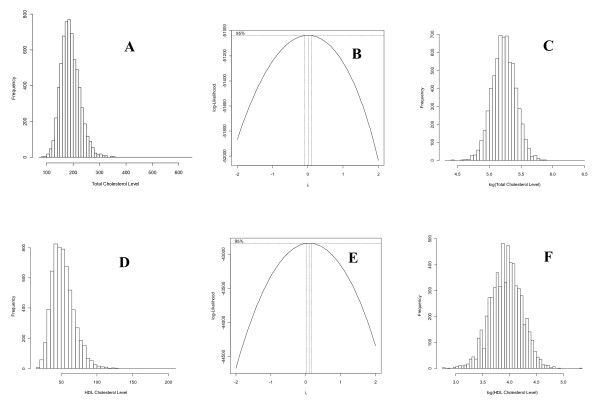
**Distributions of total cholesterol (TC) and high-density lipoprotein cholesterol (HDL-C) in original scales and in log-transformed scales**. **A**) Distribution of TC in original scale deviated from normality and had an outlier to the far right. **B**) The Box-Cox maximum likelihood analysis showed that log-transformation (λ ≈ 0) was the best transformation to achieve normality for TC. **C**) Log-transformed TC values achieved normality. One outlier to the far right was removed from the data analysis. **D**) Distribution of HDL-C in original scale deviated from normality and had some outliers to the right. **E**) The Box-Cox maximum likelihood analysis showed that log-transformation (λ ≈ 0) was the best transformation to achieve normality for HDL-C. **F**) Log-transformed HDL-C values achieved normality without serious outliers.

Original TC and HDL-C observations deviated from normality and had outliers (Figure 3A, D). The Box-Cox transformation analysis [26] implemented by the R statistical package [27] showed that the log-transformation was approximately the best transformation to achieve normality for those two traits (Figure 3B-C, E-F). One TC outlier, the highest TC value, was removed from the data analysis while no HDL-C outlier was removed. Log-transformed TC values were adjusted for blood sugar, body mass index, smoking status, and sex that had significant effects on log(TC). Age, age-squared, cholesterol treatment, and alcohol consumption were also tested for significant effects on log(TC) but were not included in the phenotypic model because they were insignificant. Log(HDL-C) was adjusted for age, age-squared, cholesterol treatment, blood sugar, body mass index, smoking status, number of cigars smoked, alcohol consumption and sex. Age was insignificant for HDL-C but was included because age-squared was nearly significant (p < 0.0543). Single-locus and epistasis effects for both traits were tested using the extended Kempthorne model that allows Hardy-Weinberg disequilibrium and linkage disequilibrium [13]. For each SNP, three effects were tested, genotypic, additive (A) and dominance (D) effects. For each SNP pair, five effects were tested, two-locus genotypic effect, A × A, A × D, D × A, and D × D epistasis effects. The EPISNPmpi parallel computing program [28] with a modification to implement a generalized least squares (GLS) analysis to account for sib correlations [29] was used to implement the statistical tests of single-locus and pairwise epistasis effects. For single-locus tests, p = 7.2 × 10^-8 ^was used as the threshold p-value to declare genome-wide significance [30]. To assess genome-wide significance of pairwise epistasis results, we used 5% type-I error with the Bonferroni correction as the genome-wide significance. The 500 k SNP data was estimated to have 276,666 independent SNPs [31]. Each pairwise test was considered to have four independent tests although five effects were tested, because the two-locus marker genotypic effect was confounded with one of the four epistasis effects in reporting significant results. Therefore, the genome-wide 5% type-I errors with the Bonferroni correction was calculated as p = 0.05 [4(276,666)(276,665)/2]^-1 ^= 3.266 × 10^-13^. This 5% significance level is equivalent to "significant linkage" defined in [16]. Since the Bonferroni correction is generally considered too severe, we also reported epistasis effects reaching "suggestive linkage" with statistical evidence that would be expected to occur one time at random in a genome-wide analysis [16]. In addition to the GLS method to account for sib correlation, the genomic control (GC) method [32] was used to account for potential sub-population structures in the three generation cohort of the FHS data set. For single-locus tests, all p-values were used to estimate inflation parameters for TC and HDL-C, yielding inflation parameter estimates of 1.14 and 1.11 respectively, and test statistics from the GLS tests were then adjusted by the estimates of inflation parameters and p-values were recalculated using the GC adjusted test statistics, which resulted in fewer significant effects. For the pairwise epistasis testing, we randomly selected 50,000 p-values and test statistics from over 466 billion pairwise tests for computational efficiency. Then we estimated the inflation parameters using two samples of 50,000 data points each for TC and HDL-C, yielding inflation parameter estimates of 1.01 and 1.05 respectively. All p-values were then adjusted using the inflation parameters and such adjustments also resulted in fewer significant epistasis results. Frequency of each subclass in an epistasis effect was calculated and each subclass was required to have a minimal number of five observations. After GC adjustment, QQ plots were made to show deviations of the observed p-values from the expected p-values under the null hypothesis for significant test results for single-locus tests only. QQ plot for epistasis effects were not made because the number of p-values for epistasis tests was too large. Gene locations of significant SNPs were identified according to ENSEMBL [15] and NCBI [14] based on Build 37.0 of the human genome.

## List of abbreviations

**GWAS: **genome-wide association study. **SNP: **single nucleotide polymorphism. **TC: **total cholesterol. **HDL-C: **high-density lipoprotein cholesterol. **LDL-C: **low-density lipoprotein cholesterol. **LD: **linkage disequilibrium. **HWD: **Hardy-Weinberg disequilibrium. **A × A: **additive × additive epistasis effect. **A × D: **additive × dominance epistasis effect. **D × A: **dominance × additive epistasis effect. **D × D: **dominance × dominance epistasis effect. **GLS: **generalized least squares.

## Competing interests

The authors declare that they have no competing interests.

## Authors' contributions

LM processed the FHS data, implemented the data analysis and prepared the manuscript with YD. JY contributed to the data analysis and manuscript preparation. HBR directed the parallel computing implementation of the GWAS analysis. TT, LF and SB conducted the *In Silico *replication using the InCHIANTI cohort. YD coordinated this research and prepared the manuscript with LM. All authors read and approved this manuscript.

## Pre-publication history

The pre-publication history for this paper can be accessed here:

http://www.biomedcentral.com/1471-2350/11/55/prepub
